# Mutagenic Effects of Ribavirin on Hepatitis E Virus—Viral Extinction versus Selection of Fitness-Enhancing Mutations

**DOI:** 10.3390/v8100283

**Published:** 2016-10-13

**Authors:** Daniel Todt, Stephanie Walter, Richard J. P. Brown, Eike Steinmann

**Affiliations:** Institute of Experimental Virology, Twincore—Centre for Experimental and Clinical Infection Research, a Joint Venture between the Medical School Hannover (MHH) and the Helmholtz Centre for Infection Research, 30625 Hannover, Germany; Daniel.Todt@twincore.de (D.T.); Stephaniewalter@gmx.de (S.W.); Richard.Brown@twincore.de (R.J.P.B.)

**Keywords:** hepatitis E virus, ribavirin, RNA viruses, mutagenesis, intra-host population

## Abstract

Hepatitis E virus (HEV), an important agent of viral hepatitis worldwide, can cause severe courses of infection in pregnant women and immunosuppressed patients. To date, HEV infections can only be treated with ribavirin (RBV). Major drawbacks of this therapy are that RBV is not approved for administration to pregnant women and that the virus can acquire mutations, which render the intra-host population less sensitive or even resistant to RBV. One of the proposed modes of action of RBV is a direct mutagenic effect on viral genomes, inducing mismatches and subsequent nucleotide substitutions. These transition events can drive the already error-prone viral replication beyond an error threshold, causing viral population extinction. In contrast, the expanded heterogeneous viral population can facilitate selection of mutant viruses with enhanced replication fitness. Emergence of these mutant viruses can lead to therapeutic failure. Consequently, the onset of RBV treatment in chronically HEV-infected individuals can result in two divergent outcomes: viral extinction versus selection of fitness-enhanced viruses. Following an overview of RNA viruses treated with RBV in clinics and a summary of the different antiviral modes of action of this drug, we focus on the mutagenic effect of RBV on HEV intrahost populations, and how HEV is able to overcome lethal mutagenesis.

## 1. Introduction

Hepatitis E virus (HEV) was first described as novel agent responsible for enterically transmitted non-A, non-B hepatitis by Reyes and colleagues in 1991 [1]. This was 35 years after the first documented epidemic outbreak (1955–1956) of a retrospectively identified HEV infection—transmitted via the fecal–oral route—in New Delhi, India [2].

HEV is a nonenveloped single-stranded RNA virus with a 7.2 kb genome of positive orientation. Three open reading frames (ORFs) encode for: (1) the nonstructural proteins (ORF1), comprising a methyltransferase, a papain-like cysteine protease, a helicase, and an RNA-dependent RNA polymerase (RdRp), connected by a Y-domain and a hypervariable region (HVR); (2) the capsid protein (ORF2); and (3) small proteins whose functions are not yet completely understood (ORF3) [3]. The viral subgenomic RNA is comparable to mammalian mRNAs, flanked by a 5′-methylguanine cap and a 3′-poly(A) tail. HEV has recently been taxonomically reassigned to the genus *Orthohepevirus* in the family of *Hepeviridae* [4]. Differences in the sequences of isolates led to the current classification into seven genotypes, four of which infect humans. HEV-1 and HEV-2 (i.e., genotypes 1 and 2) are solely human pathogens and are mainly transmitted orally by feces-contaminated drinking water. These genotypes are endemic in Africa, southeast Asia, and Mexico, while the zoonotic genotypes 3 and 4 are predominantly found in northern America, Europe, and northern Asia as summarized by the *Hepeviridae* Study Group of the International Committee on the Taxonomy of Viruses [4] and others [5].

An infection with HEV is usually self-limiting, causing arthralgia, flu-like myalgia, vomiting, and symptoms characteristic of hepatitis like jaundice and itching [6]. Progression to chronicity is generally described for pregnant women and immunosuppressed individuals, such as patients recovering form solid organ transplantation [7]. Data for HIV-coinfected patients are contradictory and still under discussion, as extensively reviewed by Debes et al. [8,9]. According to the World Health Organization (WHO), each year more than 20 million individuals are newly infected with the HEV [10]. With more than three million symptomatic cases of HEV infection reported worldwide each year and about 70,000 HEV-related deaths [6], HEV must be reconsidered to be a major global health burden, with appropriate resources redirected toward effective control and eventual eradication [11,12]. Recently, studies reporting extrahepatic manifestations of HEV have accumulated, detailing potential connections between HEV infection and neurological disorders, including Guillain-Barré syndrome [13,14,15,16,17,18,19].

Ribavirin (RBV) is a broad-spectrum antiviral agent with numerous clinical applications against viral pathogens; it is currently the only treatment option for chronically infected HEV patients. Several publications have documented the emergence of single-nucleotide variants (SNVs) in viral genomes that cause either reduced RBV sensitivity or RBV resistance [20,21,22,23]. Recent studies also indicate HEV acquired mutations under RBV therapy that decreased the sensitivity to RBV treatment regimes in vitro and, most importantly, in vivo [24,25,26].

In this article, we start with an overview of selected RNA viruses that are or have been clinically treated with RBV, and summarize this drug’s different antiviral modes of action. The second part focuses on the mutagenic effect of RBV on HEV intra-host populations and how HEV is able to overcome the lethal mutagenesis induced by this guanosine analog.

## 2. RNA Viruses and Ribavirin

**Figure 1:**
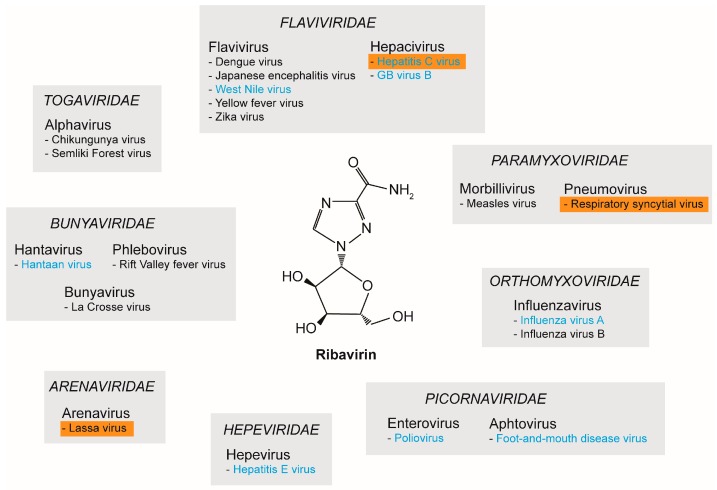
Antiviral properties of ribavirin (RBV) against RNA viruses. The broad-spectrum antiviral properties of RBV have been reported for several RNA viruses. Depicted is a selection of the different viral families and the respective genus and species. Viruses for which RBV was clinically approved are highlighted with an orange box. Viruses for which lethal mutagenesis or increased mutation rate was proposed as a possible RBV mechanism are indicated in blue.

In 1972, RBV was described as a broad-spectrum antiviral against several DNA and RNA viruses [27]. Since then, numerous studies have reported on the in vitro antiviral properties of RBV. Figure 1 provides an overview of a selection of RNA viruses against which RBV was shown to be active: hepatitis C virus (HCV, *Flaviviridae*), dengue virus (DENV, *Flaviviridae*), respiratory syncytial virus (RSV, *Paramyxoviridae*), influenza A and B virus (*Orthomyxoviridae*), chikungunya virus (CHIKV, *Togaviridae*), poliovirus (*Picornaviridae*), Hantaan virus (*Bunyaviridae*), and Lassa virus (*Arenaviridae*) [28,29] (Figure 1). For further reading we would like to refer to other reviews like [29,30,31].

Studying multiple viruses from the family *Flaviviridae*, Crance et al. investigated the in vitro antiviral properties of RBV against 11 flaviviruses including DENV, Japanese encephalitis virus (JEV), and yellow fever virus (YFV). Inhibition of virus replication was observed for all tested flaviviruses [32]. Furthermore, effectiveness of RBV could be confirmed in vivo for YFV using a hamster model by administering early upon infection [33,34]. However, these effects could not be confirmed in a nonhuman primate model [33]. Therefore, further studies are required to evaluate the possible application of RBV as a treatment option for YFV. Here, the dosage as well as the time points of treatment represent the major hurdles, which need to be overcome [33]. Additionally, hemorrhagic fever-causing viruses, which are categorized into the families of *Arena*-, *Bunya*-, and *Togaviridae*, were demonstrated to be susceptible to inhibition by RBV (Figure 1). For example, for Lassa virus the antiviral efficiency of RBV was proven both in vitro and in vivo in guinea pigs and monkeys [35,36]. Hantaviruses (i.e., Hantaan virus) and phleboviruses (i.e., Rift Valley fever virus, RVF) were also shown to be susceptible to RBV treatment [35]. In a mouse model for Hantaan virus, an increase of survival and milder signs of disease were described [35]. In experimental RVF infections of mice and hamsters, RBV led to a prevention of death, delay of death, or the onset of milder symptoms, depending on the time point of administration [35]. In general, higher doses of RBV were needed to inhibit flaviviruses compared to arena-, bunya-, and hantaviruses [33]. For CHIKV, RBV also demonstrated antiviral effects, although to a lesser extent when compared to interferon-α (IFN-α). Nevertheless, a synergistic effect of RBV and IFN-α could be demonstrated in vitro [37,38]. Moreover, the antiviral properties of RBV could be shown for members of the *Picornaviridae*: both foot-and-mouth disease virus (FMDV) [39,40] and poliovirus (PV) [41] were inhibited by RBV (Figure 1).

While displaying broad antiviral activity against a wide range of RNA viruses, clinical data on the application of RBV are still limited and restricted to only a few viruses. Initially, RBV was considered as a treatment option for influenza A and B virus infections. However, clinical trials showed inconclusive data; although some studies reported an improvement of symptoms of influenza virus infection, results were generally inconsistent [42,43]. Due to lack of conclusive data from clinical trials, coupled with the development of alternative antiviral therapies, RBV has never been approved for the treatment of influenza virus. Nonetheless, Lassa virus, HCV, and RSV are prominent examples of viruses for which RBV has received approval as an antiviral compound for clinical application [44]. RBV was shown to be effective in treating patients suffering from Lassa fever [45] and can be administered orally, intravenously, or as pre- or post-exposure prophylaxis [46]. In 1998, RBV was approved by the Food and Drug Administration (FDA) as a treatment option for HCV [47] and was, in combination with pegylated IFN-α, the standard treatment for chronic HCV infection for over two decades [48]. It has been shown that after failure of a monotherapy with IFN-α alone, a combination therapy with RBV is more effective than subsequent repetition of IFN-α monotherapy [49]. However, the sustained virological response (SVR) rates varied among genotypes, and dual therapy was associated with severe side effects [48]. Nowadays, RBV is no longer the standard-of-care anti-HCV therapy, and has been replaced by direct-acting antivirals (DAAs). Initial trials for the treatment of RSV infection showed a reduced duration of hospitalization and requirement of mechanical ventilation [50,51]. A routine use of RBV in RSV-infected children is not recommended; however, treatment can be considered for individual cases [50]. 

Taken together, since its first description as an antiviral in 1972, RBV has been shown to be active against a broad range of RNA viruses. However, due to limited clinical trial data supporting its in vivo efficacy, clinical applications are currently limited to a minority of viruses.

## 3. Multiple Modes of Action for Ribavirin

**Figure 2:**
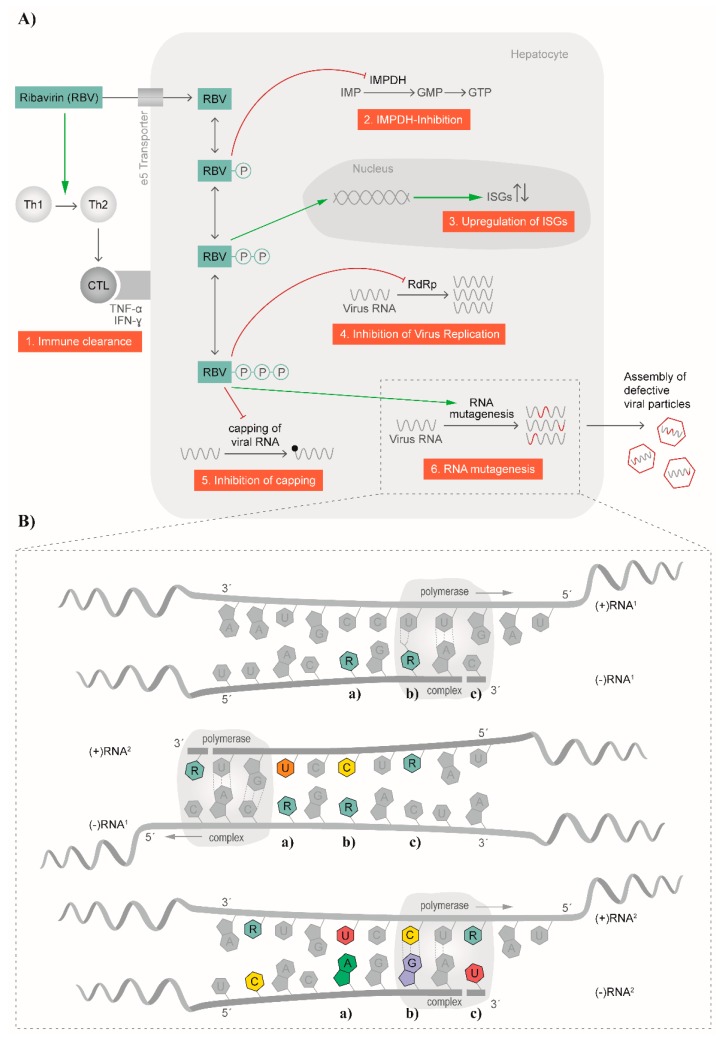
Mode of action of ribavirin. (**A**) Several antiviral mechanisms for ribavirin have been proposed and are depicted schematically. Among these are effects on the immune clearance, inhibition of inosine monophosphate dehydrogenase (IMPDH), influence on interferon-stimulated genes (ISGs), inhibition of viral replication, inhibition of capping, and RNA mutagenesis; (**B**) The mutagenic effect on RNA is visualized in more detail. In altering the synthesis of (−)RNA and (+)RNA, RBV is randomly incorporated in the nascent strands and subsequently leads to transition events causing C–U (**a**), U–C (**b**) and G–A (**c**) or A–G substitutions. CTL: cytotoxic T lymphocyte; Th1: T helper cell, type 1; Th2: T helper cell, type 2; TNFα: Tumor necrosis factor alpha; INFγ: interferon gamma; IMP: inosine monophosphate; GMP: guanosine monophosphate; GTP: guanosine triphosphate.

The broad antiviral effect of RBV against numerous RNA viruses suggests different modes of action for the molecule; indeed, several antiviral mechanisms have been described in the past [29,52] and are summarized in Figure 2A. Among the indirect mechanisms, a T-cell-mediated effect was described for HCV (Figure 2A). Here, the balance of T helper cells was changed by switching from a T helper type 2 phenotype to a T helper type 1 [29]. In a study by Hultgren et al., an inhibition of in vitro T-cell proliferation as well as a change in secreted cytokines was observed [53]. Simultaneously, alanine transaminase (ALT) levels in serum were reduced with no change in HCV titers [53]. Furthermore, an early switch of a T helper type 1 immune response to a T helper type 2 immune response was associated with disease progression and the development of chronicity [54]. Thus, RBV restored the T helper 1 phenotype needed for balanced expression and secretion of cytokines produced from type 1 and 2 T helper cells [29]. Another example where an immunomodulatory effect was described for RBV is in RSV infection. It was proposed that a T helper type 2 cytokine response initiated the cascade leading to airway hyper-reactivity, which in turn can be blocked by RBV treatment [55] (Figure 2A).

Another indirect mode of action for RBV is the inhibition of the cellular inosine monophosphate dehydrogenase (IMPDH), which was already proposed in 1973 [56] (Figure 2A). After uptake into the cell, RBV is phosphorylated to RBV mono-, di-, and triphosphate (RMP, RDP, and RTP, respectively). RMP represents a good mimic of inosine monophosphate (IMP) and thereby inhibits the synthesis of IMP to xanthosine monophosphate (XMP) by IMPDH. Consequently, no guanosine monophosphate (GMP), and subsequently guanosine triphosphate (GTP), can be synthesized. In vitro, replication of measles virus in Vero cells could be blocked by the addition of XMP, GMP, and to a lesser extent, also IMP [56], which underlines the mode of action of RMP. A linear correlation of the depletion of GTP pools and in vitro antiviral activity of RBV against human parainfluenza virus 3 and YFV was confirmed [57]. Furthermore, the addition of guanosine to cell cultures restored the antiviral activity of RBV against GB virus B (GBV-B) [58]. In contrast, in vitro experiments with Lassa virus and Hantaan virus indicated that RBV did not primarily act via depletion of GTP pools for these two viruses [59,60]. Moreover, experiments with influenza A virus showed no linear correlation of intracellular GTP pools and viral replication with increasing concentrations of RBV [61]. Additionally, the authors did not observe a complete restoration of influenza A virus replication after addition of guanosine [61]. No effect of guanosine or GMP on the antiviral effect of RBV against influenza A virus in mice could be demonstrated [62]. Overall, these data suggest that other mechanisms for the mode of action of RBV exist.

The influence of RBV on the expression of IFN-stimulated genes (ISG) is controversial in the literature. Most studies, both in vivo and in vitro, come from the HCV and RSV fields. RBV is able to increase the antiviral effects of an IFN-based therapy and restore IFN-responsiveness in HCV-infected livers [63,64,65,66]. Also, a direct, IFN-independent upregulation of ISGs has been proposed [67,68]. However, a recent study with HCV patients receiving RBV monotherapy showed a downregulation of abnormally preactivated ISGs through chromatin remodeling and modulation of histone methylation, resulting in a higher liver susceptibility to IFN by lowering the baseline expression of certain ISGs [69].

Some RNA viruses, as well as cellular mRNAs, harbor a 7-methylguanosine cap structure at the 5′ end [70]. The RBV-induced reduction of GTP pools within the cell was proposed to also have an effect on the capping efficiency of RNA viruses (Figure 2A). For example, DENV encodes for a 2′-*O*-methyltranferase at the N-terminus of the NS5 polymerase, termed NS5MTase_DV_. NS5MTase_DV_ binds GTP and catalyzes the formation of a 5′ cap structure [71]. After RBV treatment, less GTP is present and RTP was shown to compete for binding to the NS5MTase_DV_, thereby blocking the synthesis of the 5′ cap [71]. Likewise, RBV directly and strongly inhibited the viral mRNA guanylyltransferase of vaccinia virus and thus prevented capping of nascent viral RNA [72,73]. However, this mechanism is controversially discussed in literature [72,73,74,75], and not all RNA viruses display a 7-methylguanosine cap structure at the 5′ end. Therefore, this mode of action cannot account exclusively for the observed effects of RBV.

Another suggested mechanism is the direct impact of RBV treatment on the function of viral polymerases (Figure 2A,B). Here, RTP is thought to directly inhibit viral RNA replication by being recognized by the viral polymerase and thereby leading to chain termination or preventing the binding of other nucleotides important for elongation [76]. In a cell-free system, RTP was shown to inhibit the RNA polymerase of influenza A virus [77]. Moreover, inhibition of viral RNA synthesis of vesicular stomatitis virus (VSV) in the presence of RMP, RDP, and RTP was described with the triphosphorylated form being the least active [78]. This would argue against a mode of action that is based on the incorporation of RTP in the nascent viral RNA in VSV. In the same study, an inhibitory effect of RDP on La Crosse virus RNA synthesis was also reported [78]. Interestingly, Crotty et al. could demonstrate that RTP is indeed employed by PV RdRp, and that integrated RBV acts as mutagen [41]. Another example of an effect of RTP on the viral polymerase is the case of reovirus. Rankin et al. proposed that RTP binds close to the catalytic site of the transcriptase, thereby affecting the helicase function and subsequently lowering the binding affinity of viral RNA [79]. As a consequence, elongation of the viral RNA is inhibited. Interestingly, no effect on the capping activity was demonstrated [79]. The nucleotide binding site of the polymerase is highly conserved among HCV genotypes, supporting this proposed mechanism [76]. Indeed, in vitro analysis showed a minor decrease of HCV replication [52,76,80]. However, in clinical trials with RBV monotherapy, only a mild decrease of HCV replication was noticed [81,82].

In recent years, a mutagenic effect of RBV via its incorporation into newly synthesized RNA genomes, leading to viral extinction was described for several RNA viruses (Figure 2B). In contrast to DNA viruses, the major characteristic of RNA viruses is the occurrence of a cloud of related but genetically distinct variants in infected patients, often referred to as a quasispecies. However, the term “quasispecies” refers to a particular mutation–selection balance, with natural selection acting on the group rather than on the individual [83,84]. It is not simply a surrogate for genetic heterogeneity [85]. While quasispecies behavior has been demonstrated experimentally in artificially expanded poliovirus populations in infected mice [86], evidence is lacking for quasispecies’ behavior in many viruses, including HEV. These diverse intra-host viral populations are the result of the lack of proofreading activity of RdRp. However, due to this high variation, viral isolates are close to the error threshold, which would lead to reduction in viral fitness [87]. Incorporation of RBV into newly synthesized RNA genomes thereby increases the frequency of mutations in the population, pushing the virus over an error threshold and resulting in viral extinction. This mechanism of action for RBV has been described, at least in vitro, for FMDV [21], poliovirus [28], HCV [88], GBV-B [58], Hantaan virus [89], and HEV [25,26].

Ever since the first reports by Sidwell et al. describing RBV as a broad-spectrum antiviral [27], there have been multiple discussions about its mechanisms of action. Of course one has to always keep in mind that in vitro data, where most of the proposed models arose from, cannot just be translated into in vivo situations. Remarkably enough, monotherapy with RBV is only potently effective against Lassa virus [45] and HEV [90,91]. Future studies should address questions regarding the biocompatibility of RBV and its availability in the targeted liver to investigate if intracellular concentrations can account for the different proposed mechanisms—for example, to outcompete cellular nucleoside triphosphates (NTPs) for misincorporation.

In summary, several mechanisms have been postulated for RBV activity. Among these, there are indirect, immunomodulatory mechanisms and effects on IMPDH. Furthermore, mechanisms on the virus itself were described by inhibition of the capping efficiency, the viral polymerase, and a mutagenic effect on newly synthesized RNA genomes.

## 4. Hepatitis E Virus as Intra-Host Viral Populations

RNA viruses do not exist as a clonal population of genomes within the infected host, but rather diversify into a swarm of related but non-identical genome sequences [83]. This heterogeneous viral population—also referred to as mutant cloud, mutant swarm, or mutant spectra—is capable of better adapting to changing environmental conditions and rapidly evolving, during passage from host to host, due to its high heterogeneity. The concept of quasispecies was mainly developed by Manfred Eigen and Peter Schuster [92]. By demonstrating viral heterogeneity for FMDV [93,94] and VSV [95,96] Domingo and colleagues and Holland and colleagues were the first to extrapolate this concept to virology [84,97].

These viral populations are the product of very high replication rates found in RNA viruses, coupled with a lack of an RdRp proofreading function. For HCV, it is estimated that between 10^11^ and 10^12^ new virions are produced in one infected individual per day [98,99]. Estimates for HEV do not currently exist, although comparably high replication rates can be assumed. There is data on 3′-end repair mechanisms identified in small RNA viral polymerases [100]. In coronaviruses, for example, a 3′-to-5′ exoribonuclease (ExoN) domain within the nonstructural protein 14 was identified as being essential for high-fidelity replication [101,102]; for HCV, pyrophosphorolytic and NTP-mediated nucleotide excision activity of the NS5B RdRp have been described as viral mechanisms for removing misincorporated bases [103,104]. Despite these reports, most RNA viruses, and most likely also HEV, do not have any real proofreading capability, causing an error-prone replication of viral genomes. Together with the short generation times, this results in highly diverse intra-host populations [96].

As expected, HEV also exists as a heterogeneous population within infected individuals [25,26,105,106,107]. Early publications relied on the classical tools for detecting diversification of viral genomes, including restriction fragment length polymorphism (RFLP) and haplotype profiling [105,106] or clonal sequencing [107] to characterize HEV intra-host diversity. Recently, next-generation sequencing (NGS) methods have been utilized to study the distribution of SNVs in HEV genomes over time [25,26].

HEV and most other RNA virus populations exist in close proximity to the so-called genomic error threshold, which defines a maximum error rate that still guarantees the maintenance and transmission of the genetic information of the master sequence [83,84]. A replication and, most importantly, mutation rate beyond this extinction threshold causes a sharp reduction in the efficiency of transmission of the genetic information contained in the population master sequence to the next generation of viral progeny, a phenomenon sometimes referred to as error catastrophe [108]: the majority of genomes in the population are nonfunctional. Broad-spectrum antiviral agents like RBV can cause increased mutation rates, and potentially can result in the extinction of the virus population in a process called lethal mutagenesis [108]. However, the mutated viral intra-host populations can acquire mutations accounting for drug resistance or decreased sensitivity to RBV as a direct consequence of the boosted complexity of the mutational spectra. This has been shown for several viruses like HCV [20], FMDV [21], PV [22], Sindbis virus [23], and also for HEV [24,25,26]. The dynamics of HEV populations in patients under RBV therapy is not fully understood, but recent studies and reports from other RNA viruses point to a dichotomy of opposing outcomes resulting from RBV therapy: RBV-induced lethal mutagenesis resulting in viral extinction versus the accumulation of mutations beneficial to the virus in the population, which can lead to therapeutic failure [25,26].

As a consequence of the emergence of RBV-resistant mutations and subsequent treatment failure, clinicians could draw back on combination therapies to overcome or avoid this phenomenon. Possible combinations are one mutagen and a conventional antiviral drug or using several RNA mutagens in combination or sequence as proposed by Perales and Domingo [109,110,111].

## 5. Hepatitis E Virus and Mechanisms of Ribavirin Action

HEV is one of the pathogenic viruses that can currently only be treated with RBV as an off-label drug. IFN-α as an alternative therapy has been evaluated in small patient cohorts with limited success and considerable side effects [112,113]. In addition, in vitro data suggests careful assessment of IFNs when treating HEV [114,115]. Considering high mortality rates of over 20% for genotype-1-infected pregnant women [116,117], the urgent need for extensive research in the field of novel anti-HEV treatment regimens is required. Patients who fail to achieve sustained virological responses after RBV therapy for HEV have no further treatment options: this is particularly of importance in a solid organ transplant setting, as a reduction of immunosuppression beyond a certain level will lead to the rejection of the allograft [118,119], and hepatitis caused by HEV cannot be impeded.

Recently, two independent studies were able to correlate RBV treatment failure with the emergence of novel single-nucleotide variations in the viral genome during treatment [25,26]. Both research groups identified a variant previously described, G1634R [24], as well as other new variants, K1383N, D1384G, K1398R, V1479I, and Y1587F, all in the polymerase region of ORF1. In addition, Todt et al. also determined nine additional SNVs in ORFs 2 and 3 [26]. In both studies, K1383N mutations emerged in several patients; additionally, an overall increase in viral intra-host heterogeneity could be shown [25,26]. The authors demonstrated significant increases in the number of sites exhibiting SNVs, synonymous as well as nonsynonymous, in viral populations after the first administration of RBV in nine patients. This phenomenon was observed for all ORFs of the HEV genome. Interestingly, this increase in heterogeneity was reversible with a decline in the number of SNV sites when RBV treatment was stopped. Strikingly, none of the described variants that became dominant in the viral populations under treatment resulted in a decreased sensitivity to RBV when cloned into an HEV subgenomic reporter replicon in tissue culture. Only G1634R mutations altered the viral replication efficacy, increasing replication rates [24,26], while RBV sensitivity was unmodified [24,26,120]. Why RBV treatment fails in some patients, while others are able to clear the virus under RBV monotherapy, remains an open question.

RBV has been shown to block HEV replication through a depletion of cellular GTP pools in cell culture model systems [121], in addition to the strong mutagenic effect of RBV on the HEV genome in vivo described above. RBV inhibits the IMPDH, thus causing a two-fold reduction of the intracellular GTP pools and increasing CTP and UTP concentrations at the same time [122,123]. HEV genome replication is a cyclic process of alternating synthesis of negative-strand RNA and positive-strand RNA [124]. During the replication process, the extrinsically administered, RTP is randomly incorporated into the nascent negative-stranded RNA as a result of pairing with either of the pyrimidine bases cytidine or uracil (Figure 2B, upper panel). This negative-stranded antigenome RNA then serves as a template for subsequent production of positive-stranded genomic RNA. The RdRp subsequently incorporates, again randomly, a cytidine or uracil at RBV residues located in the antigenome template (Figure 2B, middle panel). These stochastic incorporations lead to nucleotide substitutions in the newly synthesized viral genomes. Additionally, RBV will also be incorporated in the positive-stranded RNA genome, leading to increased amounts of replication-defective viral genomes packaged into the capsid, ultimately leading to an increase in frequency of replication-defective virions. Additionally, new antigenome templates can be produced from defective positive-stranded genomes, so misincorporations are amplified in the replication process (Figure 2B, lower panel). This results in the fixation of transitional substitutions in nascent RNAs. Transitional purine-to-purine (G<>A) or pyrimidine-to-pyrimidine (C<>T) nucleotide substitutions are preferentially enriched during RBV monotherapy, leading to the observed synonymous exchanges as well as to the amino acid replacements favorable for the survival of the viral population [25,26]. Whether RBV also inhibits the HEV methyltransferase comparably to the direct inhibition of the vaccinia virus guanylyltransferase (see above), or if the RTP is incorporated as a cap analog [72,73] (thus impacting correct translation) has not been investigated yet. 

The mutagenic effect of RBV-based therapy can have divergent effects on HEV populations, which may impact the therapy success. On the one hand, RBV increases the mutation rate in the viral genome, driving the population towards its extinction threshold. In contrast, the increased variability in the viral population can result in selection of variants with improved replication fitness which become dominant in the viral population and are associated with therapeutic failure. These advantageous variants could be (i) a downregulation of the replication machinery, thus preventing the accumulation of more mutations, as shown from in vitro data when reverse engineering the K1383N variant into HEV cell culture systems [25], and (ii) an increase in viral polymerase fidelity as hypothesized by Debing et al. for the K1383N variant—a mutant with a substitution in the F1-motif of the RdRp—which could hinder the incorporation of RBV into the viral genome. In fact, the lab of Esteban Domingo was able to dissect a multistep process of viral adaption to a mutagenic nucleoside analog in FMDV that led to an extinction escape by changing the fidelity of the polymerase [125].

## 6. Conclusions

HEV is a life-threatening infection when immunosuppressed individuals fail to achieve an SVR during RBV treatment. Currently, clinicians do not have alternative therapy regimens available. Recent studies have suggested that the heterogeneous viral population is able to acquire SNVs that decrease RBV sensitivity [25,26]. Their data supports a conclusion whereupon the mutagenic effect of the broad-spectrum antiviral agent leads to increased heterogeneity in the intra-host viral population introducing a race between the virus trying to gain and accumulate beneficial variations and the mutagenic potential of RBV intended to drive the virus beyond an error threshold and thus into lethal mutagenesis resulting in viral extinction.
